# Tuberculosis contact investigation results among paediatric contacts in low-incidence settings in Finland

**DOI:** 10.1007/s00431-021-04000-7

**Published:** 2021-03-02

**Authors:** Antti Kontturi, Satu Kekomäki, Eeva Ruotsalainen, Eeva Salo

**Affiliations:** 1 Doctoral Programme in Population Health, University of Helsinki, Helsinki, Finland; 2 Department of Pediatrics, Children’s Hospital, University of Helsinki and Helsinki University Hospital, Helsinki, Finland; 3 Division of Infectious Diseases, Inflammation Center, Helsinki University Hospital, Helsinki, Finland

**Keywords:** Tuberculosis, Vulnerable young children, Paediatric TB contact tracing

## Abstract

Tuberculosis (TB) risk is highest immediately after primary infection, and young children are vulnerable to rapid and severe TB disease. Contact tracing should identify infected children rapidly and simultaneously target resources effectively. We conducted a retrospective review of the paediatric TB contact tracing results in the Hospital District of Helsinki and Uusimaa from 2012 to 2016 and identified risk factors for TB disease or infection. Altogether, 121 index cases had 526 paediatric contacts of whom 34 were diagnosed with TB disease or infection. The maximum delay until first contact investigation visit among the household contacts under 5 years of age with either TB disease or infection was 7 days. The yield for TB disease or infection was 4.6% and 12.8% for household contacts, 0.5% and 0% for contacts exposed in a congregate setting and 1.4% and 5.0% for other contacts, respectively. Contacts born in a TB endemic country (aOR 3.07, 95% CI 1.10–8.57), with household exposure (aOR 2.96, 95% CI 1.33–6.58) or a sputum smear positive index case (aOR 3.96, 95% CI 1.20–13.03) were more likely to have TB disease or infection.

*Conclusions*: Prompt TB investigations and early diagnosis can be achieved with a well-organised contact tracing structure. The risk for TB infection or disease was higher among contacts with household exposure, a sputum smear positive index case or born in a TB endemic country. Large-scale investigations among children exposed in congregate settings can result in a very low yield and should be cautiously targeted.

**Table:** 

**What is Known:** • *Vulnerable young children are a high priority in contact tracing and should be evaluated as soon as possible after TB exposure*
**What is New:** • *Prompt investigations for paediatric TB contacts and early diagnosis of infected children can be achieved with a well-organised contact tracing structure* • *Large-scale investigations among children exposed in congregate settings can result in a very low yield and should be cautiously targeted*

**Supplementary Information:**

The online version contains supplementary material available at 10.1007/s00431-021-04000-7.

## Introduction

In Europe, highly infectious pulmonary TB (pTB) cases are concentrating to the working age immigrant population and TB prevention remains a major public health concern [1]. Young children are especially vulnerable and eventually 30–80% of children with household exposure are infected with TB [2, 3]. Without preventive treatment, children under 5 years of age are especially prone to progression from TB infection to disease, and the risk is highest immediately after primary infection [4, 5].

Contact tracing is an imperative part of TB control [6]. Capturing and treating recently acquired infections decreases the pool of future TB cases especially in low-incidence countries [7, 8]. Because young age is a pivotal risk factor, TB yield among the youngest exposed provides important insight into the swiftness of contact tracing. Childhood TB infections are also sentinel events indicating ongoing transmission in the community but national TB registers capture only active TB [9]. Large-scale investigations can result in low yield and resources should be targeted effectively [10]. Recent publications concerning paediatric contact investigations in a low-incidence setting in Europe are scarce and few address the timing of investigations [4].

Our aim was to evaluate the contact tracing results and identify risk factors for TB disease or infection among children exposed to TB in the Hospital District of Helsinki and Uusimaa (HUS) area in Finland.

## Materials and methods

### Study population

We conducted a retrospective cohort study and reviewed the records of all paediatric contacts exposed to TB in the HUS area from 2012 to 2016 and their respective index cases. HUS includes 24 member municipalities with approximately 1.7 million residents [11]. Currently, approximately 250 TB cases emerge annually in Finland. Of these, a third occur in the HUS area [12]. All TB contact tracing in the area is supervised by the HUS epidemiological unit and all paediatric TB patients are treated in the same public tertiary hospital.

We identified the index cases and contacts from the epidemiological unit records and collected retrospective data concerning the patient characteristics, exposure, investigations and results from the medical records. Contacts under the age of 16 at the time of exposure were listed as paediatric. Older siblings aged 16 to 17 were included if they underwent investigations together with their younger siblings in the paediatric clinic. We excluded contacts who were exposed outside the HUS area or deemed unexposed at the first contact investigation visit (i.e. no contact with the index case).

### Ethics statement

The ethics approval for the study was given by the Research Ethical Committee of the HUS.

### Definitions

The exposed children were classified retrospectively from the epidemiological unit records and medical records. The exact duration of exposure was not retrospectively available; however, the national TB program in Finland includes national guidelines for TB contact tracing that dictates contacts who are included in contact investigations. The guidelines in place during the whole retrospective study period were published in 2011 by the Finnish Institute for Health and Welfare (THL). Briefly, in the guidelines, an index case is the initially identified case of new or recurrent TB. An index case with a sputum smear-positive or cavitary pTB or smear-positive secreting extrapulmonary TB is considered highly infectious. Primary investigations are recommended to all who have been exposed to highly infectious index case in the same household or enclosed space over eight cumulative hours. Additionally, primary investigations are recommended to children under 5 years of age who have been exposed to any pTB index case in the same household or enclosed space over eight cumulative hours, or to a highly infectious index case repeatedly in an enclosed space for less than eight cumulative hours. Based on the records, we classified the exposure of the children as household (i.e. exposure in the same household), congregate (i.e. exposure in a school or reception centre) or other (i.e. all other contacts included in contact investigations).

The outcome was classified as diagnosed by the treating physician at the last contact investigation visit: TB disease (i.e. confirmed or clinically diagnosed case of TB disease), TB infection (i.e. diagnosed TB infection without signs of disease), no infection or unknown (i.e. incomplete contact investigations or data unavailable). We also reviewed the medical records of the exposed children since the last contact investigation visit for a period of ≥ 2 years after the exposure to identify TB cases diagnosed in the HUS area outside the contact investigations.

The Bacille Calmette-Guérin (BCG) vaccination status was classified from the medical records (i.e. vaccination chart, parent reported or BCG scar). Based on previous literature, we evaluated if the patient had any medical conditions considered to increased risk for progression to disease (e.g. HIV, malignancy or immunosuppressive therapy) [13]. We determined the contact tracing delay as the interval in days from the diagnosis of index case (i.e. first date of TB treatment) to the date of first TB contact investigation visit.

### Statistical analysis

We used Mann-Whitney *U* or Kruskal-Wallis and two-tailed Fisher’s exact or chi-square tests for the comparison of continuous and categorical data, respectively. Binary logistic regression was used to examine potential risk factors for infection. Factors for univariate analysis (odds ratios, ORs) were selected based on previous literature [13]. Age, gender and factors with significant association were selected for multivariable analysis (adjusted odds ratios, aORs). Because of the known association between cavitary disease and sputum smear positivity, these factors were combined so that a reference group without cavitation and smear negative could be used in the multivariable model [14]. A *p*-value less than 0.05 was considered statistically significant. Data was analysed with SPSS Statistics (Version 24, IBM Corporation, Armonk, NY, US).

## Results

**Fig. 1:**
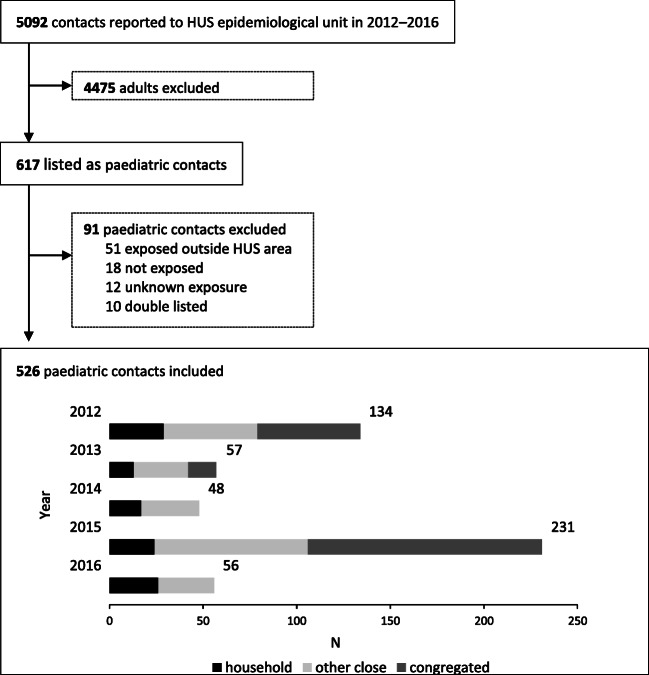
Flowchart of data collection and the annual number of contacts by exposure

There were 5092 listed TB contacts of whom 526 (10.3%) were included to the data analysis together with their 121 index cases (Fig. 1). The characteristics, contact tracing delays and outcomes of the exposed children are presented in Table 1, and the characteristics of the index cases in Table 2.

**Table 1:** The characteristics, contact tracing delays and outcome of paediatric TB contacts exposed to TB in HUS area 2012–2016.

		Total	Exposure group			Difference between groups (*p*)	
			Household (1)	Other (2)	Congregate (3)	1 vs. 2	1 vs. 2 vs. 3
Contact	*N*	526	109 (20.3)	221 (41.1)	196 (36.4)	N/A	N/A
	Age, years					0.32	< 0.0001
	Median	6.57	5.05	4.03	12.02		
	IQR	2.5–13.5	2.2–10.8	1.7–10.0	4.0–14.3		
	Age group, years					0.52	< 0.0001
	< 5	224 (42.6)	51 (46.8)	118 (53.4)	55 (28.1)		
	5–9	105 (20.0)	28 (25.7)	48 (21.7)	29 (14.8)		
	≥ 10	197 (37.5)	30 (27.5)	55 (24.9)	112 (57.1)		
	Sex					0.77	0.03
	Male	273 (51.9)	63 (57.8)	124 (56.1)	86 (43.9)		
	Female	249 (47.3)	46 (42.2)	98 (44.1)	105 (53.8)		
	Unknown	4 (0.8)	0	0	4 (2.1)		
	Birth					0.001	< 0.0001
	Native	411 (78.1)	85 (78.0)	198 (89.6)	128 (65.3)		
	Immigrant	106 (20.2)	24 (22.0)	20 (9.0)	62 (31.6)		
	TB endemic country^a^	96 (18.3)	19 (17.4)	15 (6.8)	62 (31.6)	0.003	< 0.0001
	Unknown	9 (1.7)	0	3 (1.4)	6 (3.1)		
	BCG					< 0.0001	< 0.0001
	Yes	234 (43.5)	87 (79.8)	120 (54.3)	27 (13.8)		
	Among < 5 age group^b^	92 (48.7)	41 (80.4)	43 (40.2)	8 (25.8)		
	No	121 (23.0)	16 (14.7)	77 (34.8)	28 (14.3)		
	Unknown	171 (32.5)	6 (5.5)	24 (10.9)	141 (71.9)		
	Delay, days						
	Median	18	10	14	30	0.02	< 0.0001
	IQR	10–35	4–19	8–25	22–42		
	≤ 7 days^b^	102 (21.0)	51 (46.8)	46 (22.5)	5 (2.9)	0.04	< 0.0001
	Among < 5 age group^b^	64 (30.6)	33 (64.7)	28 (25.7)	3 (8.2)		
	Unknown	41 (7.8)	0	17 (7.7)	24 (12.2)		
Index	*N*	121	53	80	11	N/A	N/A
	Contacts/Index ratio						
	Median	2	2	2	7	N/A	N/A
	Range	1–94	1–5	1–14	1–89		
	Relation to contact					N/A	N/A
	Parent	61 (11.6)	56 (51.4)	5 (2.3)	0		
	Sibling	16 (3.0)	16 (14.7)	0	0		
	Grandparent	82 (15.6)	18 (16.5)	64 (29.0)	0		
	Aunt/uncle	35 (6.7)	7 (6.4)	28 (12.7)	0		
	Other	332 (63.1)	12 (11.0)	124 (56.1)	196 (100.0)		
Outcome	TB disease or infection	34 (6.5)	19 (17.4)	14 (6.3)	1 (0.5)	0.003	< 0.0001
	TB disease	9 (1.7)	5 (4.6)	3 (1.4)	1 (0.5)	0.13	0.07
	< 5 years	3 (1.3)	2 (3.9)	1 (0.8)	0	0.22	0.19
	Immigrant	5 (4.7)	4 (16.7)	0	1 (1.6)	0.11	0.02
	TB infection	25 (4.8)	14 (12.8)	11 (5.0)	0	0.02	< 0.0001
	< 5 years	7 (3.1)	1 (2.0)	6 (5.1)	0	0.68	0.20
	Immigrant	6 (5.7)	5 (20.8)	1 (5.0)	0	0.20	< 0.001
	No infection	431 (81.9)	87 (79.8)	189 (85.5)	155 (79.5)		
	Received prophylaxis	41 (7.8)	14 (12.8)	26 (11.8)	1 (0.5)	N/A	N/A
	Unknown	61 (11.6)	3 (2.8)	18 (8.1)	40 (20.4)		
	Outside HUS^c^	23 (4.4)	0	13 (5.9)	10 (5.1)		
	Lost to follow-up	36 (6.8)	3 (2.8)	5 (2.3)	28 (14.4)	0.99	< 0.0001
	No data	2 (0.4)	0	0	2 (1.0)		

Note: Categorical data are *N* (%)

*N/A* not applicable, *BCG* Bacille Calmette-Guérin vaccination, *IQR* interquartile range

^a^Born in a high TB incidence (≥ 50/100,000) country

^b^Percentage out of those with BCG or delay data available

^c^Contact investigations outside the hospital district of Helsinki and Uusimaa

**Table 2:** Characteristics of the index case and relation to the outcome of the paediatric contacts

Characteristic	Total	Contacts with TB infection or disease		Difference (*p*)
		≥ 1	None	
*N*	114	21 (17.4)	93 (76.9)	N/A
Age, years				0.21
Median	39.8	38.5	40.7	
IQR	27.8–65.3	25.7–44.9	28.8–66.4	
Sex				0.99
Male	63 (55.3)	12 (57.1)	51 (54.8)	
Female	49 (43.0)	9 (42.9)	40 (43.0)	
Unknown	2 (1.7)	0	2 (2.2)	
Birth				0.16
Native	60 (52.6)	8 (38.1)	52 (55.9)	
Immigrant	54 (47.4)	13 (61.9)	41 (44.1)	
TB focus				0.46
Pulmonary	110 (96.5)	20 (95.2)	90 (96.8)	
Extrapulmonary	3 (2.6)	1 (4.8)	2 (2.2)	
Unknown	1 (0.9)	0	1 (1.1)	
Smear				0.01
Positive	(59.6)	17 (81.0)	51 (54.8)	
Negative	45 (39.5)	3 (14.3)	42 (45.2)	
Unknown	1 (0.9)	1 (4.8)	0	
Cavitation^a^				< 0.0001
Yes	29 (25.4)	14 (66.7)	15 (16.1)	
No	82 (71.9)	7 (33.3)	75 (80.6)	
Unknown	3 (2.6)	0	3 (3.2)	
MTB drug susceptibility				0.25^b^
Sensitive	94 (82.5)	17 (81.0)	77 (82.8)	
Resistant	12 (10.5)	4 (19.0)	8 (8.6)	
Mono resistant	7 (6.1)	2 (9.5)	5 (5.4)	
MDR	4 (3.5)	2 (9.5)	2 (2.2)	
XDR	1 (0.9)	0	1 (1.1)	
Unknown	8 (7.0)	0	8 (8.6)	

Note: Data on seven index cases with all contact outcomes unknown not presented. Categorical data are *N* (%)

*N/A* not applicable, *IQR* interquartile range, *MTB Mycobacterium tuberculosis*, *MDR* multidrug-resistant, *XDR* extensively drug-resistant

^a^Cavitation on chest radiograph

^b^Sensitive vs. resistant

Altogether, nine cases of TB disease and 25 cases of TB infection were diagnosed among the exposed children resulting in an overall contact investigation yield of 6.5% (34/526) for TB disease or infection. Outside the contact investigations, none of the exposed children were diagnosed with TB in the HUS area within ≥ 2 years after the exposure. The characteristics and diagnostic test results of the exposed children with TB disease or infection are presented in supplementary Table 1. The median age of the children with TB disease and infection was 10.9 years (interquartile range, IQR, 4.3–13.7 years) and 8.9 years (IQR, 3.9–12.9 years), respectively. None were identified to have a medical condition considered to increased risk for progression to disease. One child with disease (1/9) and six with infection (6/25) were diagnosed during follow-up after conversion of Tuberculin Skin Test (TST), Interferon-Gamma Release Assay (IGRA) or both. All of them were diagnosed within 4 months of the first contact investigation visit and none received preventive treatment before the diagnosis. Three of the cases diagnosed with infection during follow-up were under the age of five. At the time of diagnosis, the median TST results of the children with TB disease and infection were 18 mm (IQR, 8–19 mm) and 17 mm (IQR, 11–20 mm), respectively. None of the pTB cases were microbiologically confirmed. One child in the congregate exposure group had culture confirmed abdominal TB disease.

The maximum contact tracing delay for the children under 5 years of age with either TB disease or infections was 7 days among the household exposure group and 12 days among the other exposure group. None of the children with TB disease had severe disease forms such as meningeal or miliary disease. All made full recovery: 8/9 completed full TB treatment, and in one case, the treatment was discontinued slightly before full completion due to elevated liver enzymes. Among the exposed children with TB infection without disease, 22/25 completed full preventive treatment, one moved abroad during the treatment, and two with a multidrug resistant TB (MDR-TB) index case were followed up for over 2 years without preventive treatment or progression to disease.

The OR for TB disease or infection per exposure group is presented in Table 3. Due to low yield in the congregate exposure group, we excluded this group from further analysis. The ORs per different factors among the household and other contacts are presented in Table 4. In the univariate analysis, household exposure, BCG vaccination and birth in a TB endemic country (incidence ≥ 50/100,000) of the contact and smear positivity and pulmonary cavitation on chest x-ray of the index case were associated with TB disease or infection of the exposed children. Because BCG likely reflects birth in a TB endemic country, it was excluded from the multivariable analysis. In the multivariable analysis, household exposure (aOR 2.96, 95% confidence interval, CI 1.33–6.58) and birth in a TB endemic country (aOR 3.07, 95%CI 1.10–8.57) of the contact and smear positivity (aOR 3.96, 95% CI 1.20–13.03) of the index case were associated with TB disease or infection of the exposed children (Table 5).

**Table 3:** Unadjusted odds ratios for TB disease or infection among paediatric tuberculosis contacts according to exposure group

	OR	95% CI
Exposure		
Household	1.00	–
Other	0.34	0.16–0.71
Congregate	0.03	0.004–0.22

*OR* odds ratio, *CI* confidence interval

**Table 4:** Unadjusted odds ratios for TB disease or infection among household and other paediatric contacts according to the different factors

		OR	95% CI
Exposure	Household	2.95	*1.41–6.15*
Contact	Age group, years		
	5–9	1.00	–
	< 5	0.53	0.20–1.40
	≥ 10	1.85	0.73–4.66
	Gender, male	0.67	0.33–1.39
	BCG, yes	4.48	*1.32–15.15*
	TB endemic country^a^	4.09	*1.70–9.84*
Index	Age, years	0.99	0.97–1.01
	Gender, male	0.94	0.45–1.95
	Smear, positive	2.99	*1.12–8.02*
	Cavity, yes	4.06	*1.91–8.63*

Bold indicates statistically significant factor in logistic regression

*OR* odds ratio, *CI* confidence interval

^a^Immigrant born in a high TB incidence country (≥ 50/100,000)

**Table 5:** Adjusted odds ratios for TB disease or infection among paediatric contacts according to the different factors

		aOR	CI
Exposure Contact	Household	2.96	*1.33–6.58*
	Age group, years		
	5–9	1.0	–
	< 5	0.96	0.34–2.73
	≥ 10	1.69	0.63–4.566
	Gender, male	0.67	0.31–1.47
	TB endemic country^a^	3.07	*1.10–8.57*
Index	Infectivity		
	Smear−/cavitation−	1.0	–
	Smear+	3.96	*1.20–13.03*
	Smear−/cavitation+	1.52	0.15–15.83

Bold indicates statistically significant factor in multiple logistic regression

*aOR* adjusted odds ratio, CI confidence interval

^a^Immigrant born in a high TB incidence country (≥ 50/100,000)

## Discussion

In our retrospective contact tracing cohort from low incidence settings, approximately 10% of all listed contacts were paediatric and 4% were at the most vulnerable age of under 5 years. The European consensus is that TB contacts should be informed within a week after identification of the index case and young children are a high priority in contact tracing [13]. Since young children are prone to rapid and severe disease, those with strong exposure should be evaluated urgently. Contact tracing in the HUS area identified exposed young children quickly: most of the TB infections among the children under 5 years of age were found before progression to disease and none had severe disease forms. Most children under 5 years of age in the household exposure group had first contact investigation visit within a week and the maximum delay for those with either TB disease or infection was 7 days. Our results show that prompt investigations and early diagnosis can be achieved with well-organised contact tracing structure.

Recent paediatric contact tracing publications from low-incidence European countries are scarce [4]. A large systematic review from high-income settings found that the prevalence of active TB and latent TB infection (LTBI) was 4.7% and 16.3% among children aged ≤ 5 years and 2.9% and 18.4% among children aged 5–14 years, respectively [6]. However, the publications had substantial heterogeneity suggesting that the estimates might not be comparable with Europe. The epidemiological landscape in high-income European countries also varies: for example, in Croatia, the reported LBTI yield among close contacts under 5 years of age is much higher 26.4% [15]. In our study, the TB infection and disease yields among the exposed children under 5 years of age were much lower compared with the aforementioned figures. The relatively low yield in our study suggests that index cases in the HUS area are promptly diagnosed and isolated before transmission mostly occurs. The TB disease and infection yields among all paediatric household contacts in our study (4.6% and 12.8%) compare relatively well with those reported from a cohort of children under 18 years of age in Sweden (3.5% and 17.1%) [16]. Similar LTBI yield (13.1%) has also been reported from France among children under 15 years of age with closed room exposure time over 40 h, although TB disease was found less frequently in the French cohort (1.4%) [17]. LTBI was slightly less frequent among exposed children under 15 years of age in the UK (10%) and household contacts under 16 years of age in Germany (4.5% or 9% depending on the TST cut-off) [18, 19].

As in previous studies, the risk for TB infection or disease was higher among children with household exposure and a sputum smear positive index case [16, 17, 20]. Similar to Sweden, the risk was also higher among children born in a TB endemic country [16]. Close-contact investigations among immigrant population have been shown to be cost-effective, although foreign-born contacts are more likely to remain unidentified [21, 22]. It is likely that investigations among the immigrant community identify also old infections resulting from TB exposure abroad. In Finland, LTBI screening is only performed as a pre-BCG-immunisation investigation and approximately 20% of all immigrant TB cases are diagnosed through TB screening [23]. The date of entry or TB screening data for the immigrant cases in our study was not available. However, besides capturing TB disease early, the prompt investigations among the exposed immigrant children provided important baseline test results: 7/11 had active disease or exhibited TST and/or IGRA conversion suggesting a recent infection. Nevertheless, a more active case-detection approach among high-risk immigrant children warrants further evaluation.

The yield in the congregate exposure group was very low: the large-scale investigations among those exposed in congregate setting were mostly inefficient. We also suspect that the only TB case was coincidental and likely not related to the congregate exposure. In our national guidelines, the exposure criteria for TB investigations were essentially the same for all out-of-household contacts. Thus, it seems that the exposure risk in congregate settings is difficult to quantify and may be overestimated. Nevertheless, molecular epidemiological studies have shown that TB transmission can occur through social exposure and large school outbreaks of TB have been reported [24, 25]. Infections or the lack of them among those with the highest presumed risk can provide empirical evidence of the true infectiousness of the index case. Fruitless large-scale investigations deplete valuable contact tracing resources and might be avoided with a more cautious and targeted initial approach.

Since 2006, only children considered to have high TB exposure risk have been BCG vaccinated in Finland. Children undergoing contact investigations have evidently been at risk for TB exposure. Thus, optimally, all exposed children should have been BCG vaccinated. In Finland, majority of TB cases still occur among the older indigenous population, and TB exposure from grandparents was substantial [26]. Most of the exposed children under 5 years of age with a grandparent as an index case were non-BCG-vaccinated suggesting that this risk group of grandchildren is not comprehensively captured under the current selective BCG policy. The relatively high proportion of non-BCG-vaccinated children further emphasises the need for a rapid and effective contact tracing protocol.

### Limitations

Our retrospective study holds some limitations and results should be viewed with caution. The infectiousness of the index case and level of exposure were based on available retrospective data. Index cases were based on the epidemiological link and not molecular epidemiology, and some of the infections might be from another source. Data concerning unidentified contacts and enabling cost-benefit analysis was not available. An important factor contributing to the total delay of children exposed to TB is the diagnostic delay of the index case which was not covered in our study.

## Conclusion

Vulnerable young children should be evaluated as soon as possible after TB exposure. Prompt investigations and early diagnosis can be achieved with a well-organised contact tracing structure. Large-scale investigations after TB exposure in a congregate setting can result in a very low yield. Thus, TB investigations after such exposure should be cautiously targeted. TB disease or infection risk was higher among the children born in a TB endemic country. Timely investigations enabled identification of IGRA and TST conversions, and most immigrant cases were likely recent infections. In a risk group-based BCG vaccination policy, children with TB exposure risk from grandparents are difficult to identify for immunisation.

## Supplementary information

ESM 1 (DOCX 79 kb)

## Data Availability

The data are not publicly available due to restrictions.
