# Supplementary material for: Tuberculosis contact investigation results among paediatric contacts in low-incidence settings in Finland
Source: Eur J Pediatr. 2021 Mar 2;180(7):2185–92. doi: 10.1007/s00431-021-04000-7 (PMC8195747; doi:10.1007/s00431-021-04000-7)
Supplement: Supplementary file 1 — (DOCX 79 kb) [file 431_2021_4000_MOESM1_ESM.docx]

| **Supplementary table 1.**  Exposed children diagnosed with tuberculosis disease or infection during contact investigations. | | | | | | | | |
| --- | --- | --- | --- | --- | --- | --- | --- | --- |
| Diagnosis | Age (years) | Native-born | BCG | TST (mm) | IGRA (+/-) | Chest x-ray | Symptoms | |
| Disease | 0 | Yes | Yes | 8 | T-SPOT.TB + | L | C | |
|  | 4 | No | Yes | 18 | T-SPOT.TB + | N | F, C | |
|  | 4 | Yes | No | 20 | QFT + | I, L | – | |
|  | 6 | Yes | Yes | 6 | T-SPOT.TB + | L | F, C | |
|  | 10 | No | UNK | 17 | T-SPOT.TB + | N | F, C | |
|  | 11 | Yes | Yes | N/P | **T-SPOT.TB +** | N | C | |
|  | 12 | No | UNK | 19 | T-SPOT.TB + | N | C | |
|  | 14 | No | Yes | N/P | QFT + | I | – | |
|  | 16^1^ | No | UNK | 20 | T-SPOT.TB + | N | – | |
| Infection | 1 | Yes | Yes | **18** | **T-SPOT.TB +** | N | – | |
|  | 2 | UNK | Yes | 3 | **T-SPOT.TB +** | N | – | |
|  | 2 | Yes | No | 0 | T-SPOT.TB + | N | – | |
|  | 3 | Yes | No | N/P | QFT + | N | – | |
|  | 3 | Yes | Yes | 16 | T-SPOT.TB + | N | – | |
|  | 3 | Yes | Yes | **15** | T-SPOT.TB - | N | – | |
|  | 4 | Yes | Yes | 20 | T-SPOT.TB - | N | – | |
|  | 5 | No | Yes | 16 | QFT + | N | – | |
|  | 5 | Yes | Yes | N/P | QFT + | N | – | |
|  | 6 | Yes | Yes | 20 | T-SPOT.TB + | N | – | |
|  | 6 | Yes | Yes | **10** | T-SPOT.TB - | N | – | |
|  | 7 | Yes | Yes | 5 | T-SPOT.TB + | N | – | |
|  | 8 | Yes | Yes | 19 | T-SPOT.TB + | N | – | |
|  | 9 | Yes | Yes | N/P | QFT + | N | – | |
|  | 11 | Yes | Yes | 20 | T-SPOT.TB + | N | – | |
|  | 11 | Yes | Yes | 25 | T-SPOT.TB - | N | – | |
|  | 12 | No | Yes | 16 | QFT - | N | – | |
|  | 12 | Yes | Yes | 9 | T-SPOT.TB + | N | – | |
|  | 12 | No | Yes | 20 | T-SPOT.TB + | N | – | |
|  | 13 | Yes | Yes | 18 | T-SPOT.TB + | N | – | |
|  | 13 | Yes | Yes | 17 | T-SPOT.TB + | N | – | |
|  | 13 | No | Yes | **23** | **T-SPOT.TB +** | N | – | |
|  | 13 | Yes | Yes | 18 | T-SPOT.TB + | N | – | |
|  | 14 | No | Yes | **10** | **T-SPOT.TB +** | N | – | |
|  | 14 | No | Yes | 17 | T-SPOT.TB - | N | – | |
| NOTE.  Bold indicates conversion to positive.  +/-, positive/negative  N/P, not performed  QFT, QuantiFERON-TB Gold  TB, tuberculosis  TST, tuberculin skin test  UNK, unknown  Chest x-ray findings: I=infiltrate, L= intra-thoracic lymphadenopathy, N=normal,  Symptoms: C=prolonged cough, F=fever  ^1^ culture confirmed abdominal tuberculosis | | | | | | | |  |
